# Unexpectedly rapid evolution of mandibular shape in hominins

**DOI:** 10.1038/s41598-018-25309-8

**Published:** 2018-05-09

**Authors:** P. Raia, M. Boggioni, F. Carotenuto, S. Castiglione, M. Di Febbraro, F. Di Vincenzo, M. Melchionna, A. Mondanaro, A. Papini, A. Profico, C. Serio, A. Veneziano, V. A. Vero, L. Rook, C. Meloro, G. Manzi

**Affiliations:** 1 Università degli Studi di Napoli Federico II, Department of Earth Sciences, Environment and Resources, L.go San Marcellino 10, 80138 Naples, Italy; 2 Università degli Studi di Roma La Sapienza, Department of Environmental Biology, Piazzale Aldo Moro, 5, 00185 Roma, Italy; 3 Università degli Studi del Molise, Department of Biosciences and The Territory, Contrada Fonte Lappone, 86090 Pesche, Isernia Italy; 4 Istituto Italiano di Paleontologia Umana, Via Ulisse Aldrovandi, 18, 00197 Roma, Italy; 5 Università degli Studi di Firenze, Department of Earth Sciences, Via Giorgio La Pira, 4, 50121 Florence, Italy; 6 Liverpool John Moores University, School of Natural Science and Psychology, Byrom Street, L3 3AF Liverpool, UK

## Abstract

Members of the hominins – namely the so-called ‘australopiths’ and the species of the genus *Homo* – are known to possess short and deep mandibles and relatively small incisors and canines. It is commonly assumed that this suite of traits evolved in early members of the clade in response to changing environmental conditions and increased consumption of though food items. With the emergence of *Homo*, the functional meaning of mandible shape variation is thought to have been weakened by technological advancements and (later) by the control over fire. In contrast to this expectation, we found that mandible shape evolution in hominins is exceptionally rapid as compared to any other primate clade, and that the direction and rate of shape change (from the ape ancestor) are no different between the australopiths and *Homo*. We deem several factors including the loss of honing complex, canine reduction, and the acquisition of different diets may have concurred in producing such surprisingly high evolutionary rates. This study reveals the evolution of mandibular shape in hominins has strong morpho-functional and ecological significance attached.

## Introduction

Primates are a large group of mainly arboreal, mostly tropical mammals, ranging in body size from 30 g in Berthe’s mouse lemur (*Microcebus berthae*) to 200 kg in male gorilla. In terms of diet, primates are nearly equally variable, being adapted to feed on insects, honey, fruits, leaves, seeds, nuts, and even vertebrate meat. Such wide dietary ambit reflects in the primate mandible and teeth variation. The extent to which diet actually influences the masticatory apparatus in Primates is the subject of intense investigation. It is now well recognised that variation in both mandibular shape and body size were the primary pathways for ecological diversification in fossil, as well as in living primates^1^, with diet acting primarily at high taxonomic level, while size has stronger effects between closely related species^2^. Hominins (which include the species belonging to either *Homo* or to the so-called ‘australopiths’) make no exception to this pattern. Members of the hominin clade have been long noted for their peculiar mandible shape, with short and deep corpus (the horizontal part that bears the tooth-row), low-cusped molars, and reduced incisors and canines. This suite of features is said to allow for a diet including tough food items such as roots and seeds^3,4^, and is linked to the reduced importance of food processing by the anterior dentition, as compared to fellow apes. This habitus is common to many, but by no means to all of the australopiths^4,5^, and reached its extreme in the Early Pleistocene hominin *Paranthropus boisei*^6^, consistently with the lifestyle in the grasslands the late australopiths adapted to^7^. While living in open-habitats was common to *Homo* as well^8^, species in our own genus have smaller, thinner-enamel cheek teeth, less robust mandible and zygomatic arches^9^, reduced masticatory muscles and bite force^10^, and decreased protrusion of the dental arcade (i.e. prognathism). Most of the differences between *Homo* and the australopiths are believed to relate to the evolution of an extremely large brain in *Homo*, which is responsible for ever increasing technological abilities and, later, for the control over fire. This would have eventually released adaptive pressures on the mandible and teeth, by endowing efficient mechanical food processing before chewing^11–14^. As such, while the evolution of a mandible shape responsive to a new lifestyle and diet in australopiths should make them no different from the other primates, the robust relationship between mandible shape and diet presumably faded out in *Homo*, with the expected consequence of low evolutionary rate of change in *Homo* mandibles.

**Figure 1:**
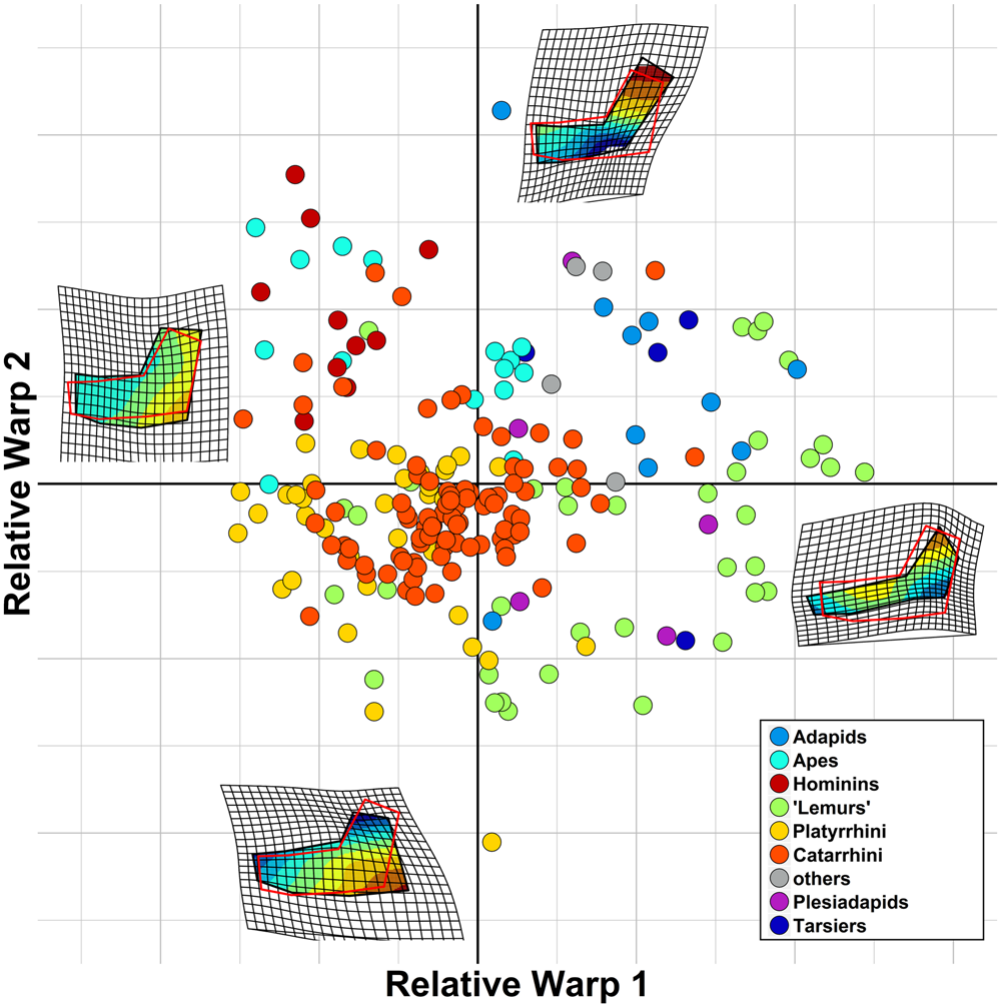
The major axes of mandibular shape variation in primates, retrieved from GMM. *Homo* and the australopiths almost exclusively occupy the upper left quadrant of the plot (purple circle). At the two extremes of both axes we reported the shape deformation associated to these axes, overimposed on the primate consensus shape (in red) and a continuous colour scale representing the mandibular areas or more intense deformation, from areas where the mandible widens compared to the consensus (in red) to areas where it compresses (in blue). The image was generated by using the R package ggplot (http://ggplot2.org/) and our own R codes.

To verify this hypothesis, we analysed mandibular shape variation in a large sample of primates, ranging from Paleogene ‘plesiadapids’ to living species, by applying geometric morphometrics (GMM) to the primate mandible under a new phylogenetic comparative method (PCM) approach^15^. We assembled a dataset of 731 primate mandible images belonging to 211 different species and built a phylogenetic tree for those. We implemented and applied the RRphylo PCM^15^, to the shape data ordinated via GMM (Fig. 1). Such method allows retrieving the rate of shape evolution for all the branches in the tree and verifies the existence of shifts in the rate of evolutionary change among clades.

## Results

**Figure 2:**
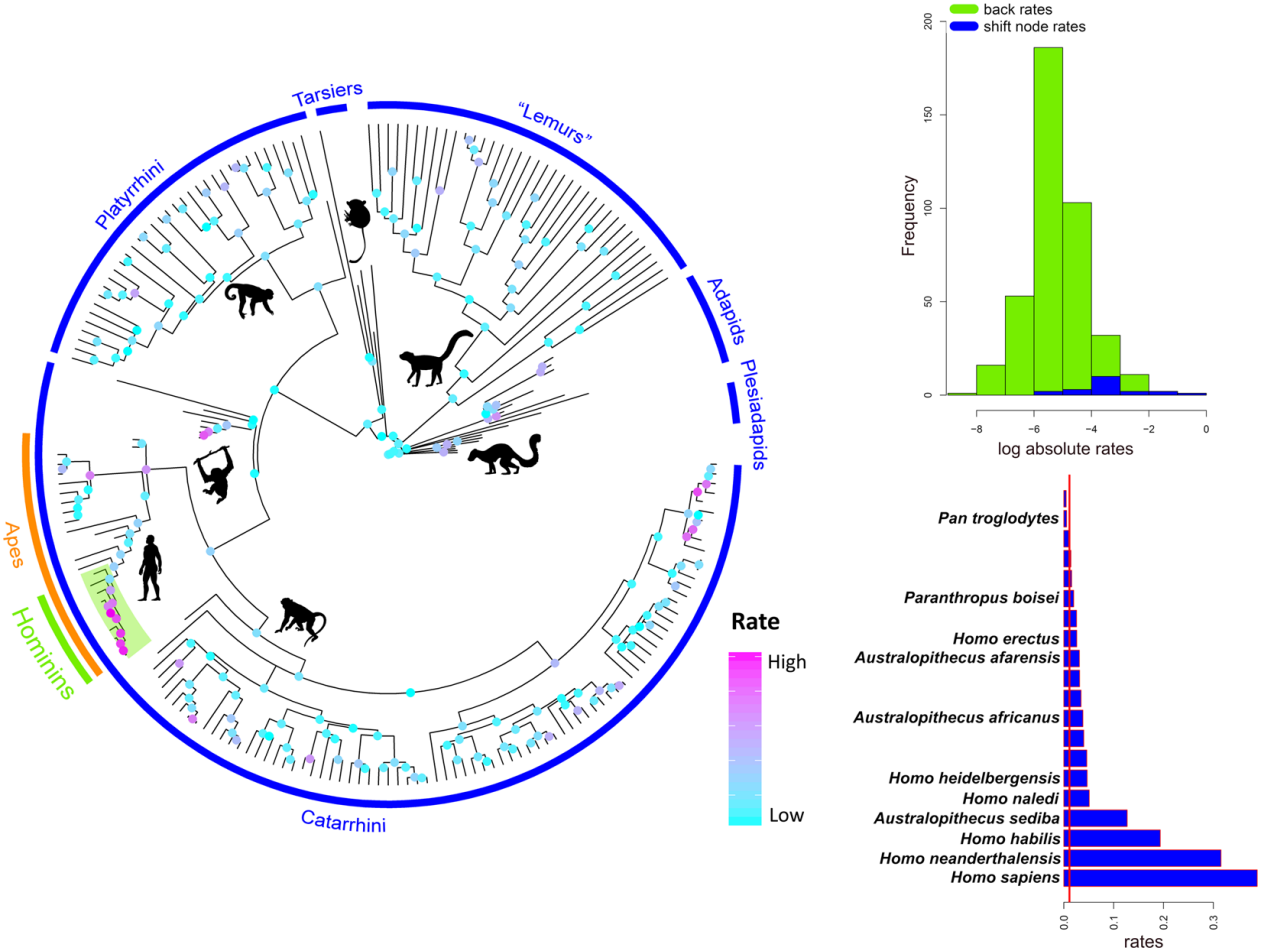
The evolutionary rates of mandible shape on the primate tree. The tree on the left reports rates computed according to phylogenetic Ridge Regression (coloured dots, scaled according to the rate value, from low = cyan, to high rates = magenta). The human clade, highlighted with a green semitransparent box, represents the only rate shift as indicated by the variable Brownian rate approach. On top right, the phylogenetic Ridge Regression rates (in absolute values) computed for the branches of the tree not belonging to the human clade (green) are contrasted to rates for the human clade (blue). On bottom right, phylogenetic Ridge Regression rates of individual branches of the human clade (in absolute value) plus the human clade sister species, the common chimpanzee, are collated in increasing rate value (blue bars), and contrasted to the average rate computed over the entire tree (the vertical red line). Bars without names correspond to internal nodes of the human clade. The image was generated by using the R package ggplot (http://ggplot2.org/) and our own R codes. Animal silhouettes were available under Public Domain license at phylopic (http://phylopic.org/), unless otherwise indicated. Specifically, clockwise starting from the bottom, *Macaca* (http://phylopic.org/image/eedde61f-3402-4f7c-9350-49b74f5e1dba/); *Homo sapiens* (http://phylopic.org/image/c089caae-43ef-4e4e-bf26-973dd4cb65c5/); *Hylobates* (http://phylopic.org/image/0174801d-15a6-4668-bfe0-4c421fbe51e8/); *Cebus* (http://phylopic.org/image/156b515d-f25c-4497-b15b-5afb832cc70c/) available for reuse under the Creative Commons Attribution 3.0 Unported (https://creativecommons.org/licenses/by/3.0/) image by Sarah Werning; *Tarsius* (http://phylopic.org/image/f598fb39-facf-43ea-a576-1861304b2fe4/); lemuriformes (http://phylopic.org/image/eefe8b60-9a26-46ed-a144-67f4ac885267/), available for reuse under Attribution-ShareAlike 3.0 Unported (https://creativecommons.org/licenses/by-sa/3.0/) image by Smokeybjb; *Plesiadapis* (http://phylopic.org/image/b6ff5568-0712-4b15-a1fd-22b289af904d/), available for reuse under Attribution-ShareAlike 3.0 Unported (https://creativecommons.org/licenses/by-sa/3.0/) image by Nobu Tamura (modified by Michael Keesey).

We found the entire hominin clade to stand out among primates, accounting for a disproportionately large share of the clade mandibular shape variation (Fig. 2). More importantly, hominins represent the only instance of (multivariate) rate shift in mandibular shape evolution in primates, either according to RRphylo, or by using the more traditional, multivariate Brownian rate variation approach (Fig. 2). This result does not depend on the tree topology and branch lengths we adopted. We produced 100 random trees where half of the node ages were allowed to vary in between the ages of their parent and descending nodes. Contemporarily, in each random tree 50% of the tips were allowed to swap position, up to three nodes from their actual position (e.g., a *Homo erectus - Homo sapiens* sister species relationship, albeit *Homo neanderthalensis* and *Homo heidelbergensis* are present in the tree, is theoretically permitted in the random trees). Despite such strong rearrangement of the topology and branch lengths, the average rate of evolution calculated for the branches of the hominin clade remains statistically higher than for the remaining part of the tree (see Figure S3). Since body size variation accounts for a large share of ecological diversification within primates^1^, and is significantly related to shape variation (see Supplementary material, and Figures S6 and S7) we also repeated the analyses after factoring out the effect of size on shape, by using the centroid size of the landmark configuration as a proxy for size. Again, only hominins stand out for having exceptionally large rates (Supplementary Figure S6).

### The direction of shape change, *Homo* and the australopiths evolved along parallel trajectories of shape change

The evolutionary rate represents the magnitude of shape change to the unit time. However, it is silent as per the direction of change. RRphylo produces vectors of regression coefficients (associated to the RW scores) describing the mandible shape change from one node in the tree to the next. Such vectors, besides their size (magnitude) have specific directions, that can be expressed in terms of the angle they form to each other, or to a specific reference. Given the indication of a rate shift in mandible shape evolution accruing to all hominins, we took the most recent common ancestor to the great apes in the tree as the reference and computed the angles between each ape species and such ancestor. Then, we partitioned the great apes in non-hominin apes (here to fore just ‘ape’ for simplicity), *Homo* species, and australopiths.

**Figure 3:**
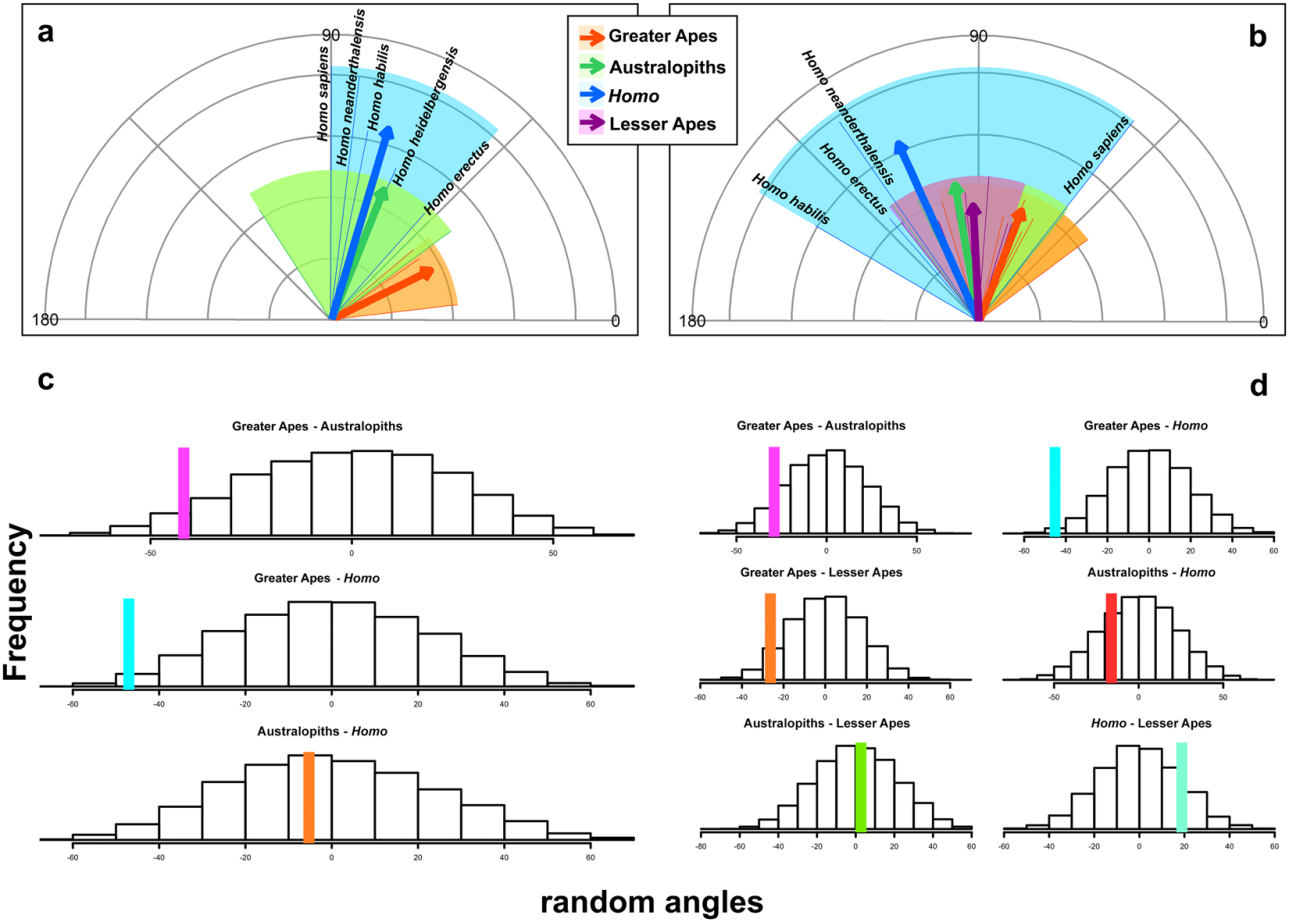
Multivariate angle comparisons among non-hominin apes, *Homo* species and the australopiths, assessed through multivariate angles between rate vectors. In (**a**) angles of *Homo*, australopiths, and non-hominin greater apes (Great Apes) are depicted starting from the common origin (the ancestor of all these species). The range of angles for each group is highlighted: *Homo*, transparent blue; Australopiths, transparent green; Great Apes, transparent orange. Vector length is proportional to actual vector size (i.e. the evolutionary rate). In (**b**) the same as with (**a**) but including lesser apes (Hylobatidae) highlighted in transparent purple. In (**c**) the angles in (**a**) are tested for significance by shuffling the rates among groups 10,000 times, real differences are indicated by the color bars. In (**d**) the angles in (**b**) are tested for significance by shuffling the rates among groups 10,000 times, real differences are indicated by color bars.

We found the mean angle of apes to the most recent common ancestor of all great apes was 26.5 degrees. For australopiths, the angle was 68.2 degrees, some 42 degrees more. For *Homo* species, the mean angle was 73.5 degrees, 47 degrees wider than apes, but only 5.3 degrees wider than the mean angle for the australopiths (Fig. 3). According to a randomization test, the difference in angles between apes and australopiths, and apes and *Homo* are both significant (p = 0.032 and p = 0.01, respectively). In contrast, the angle between australopiths and *Homo* is not significant (p = 0.43). This implies the trajectories of *Homo* and the australopiths are parallel, whereas both diverge significantly from the other greater apes’ trajectory (Table 1). The same procedure repeated with the inclusion into the analysis of the Hylobatidae (lesser apes) shows similar results, but also indicates there is no significant difference in angles between the trajectories of lesser apes and the hominins (Fig. 3b,d).

**Table 1:** Multivariate angle of evolutionary rates.

comparisons	Difference in angle	p.value			
APE_AUS	−41.74	0.06			
APE_HOM	−47.02	0.04			
AUS_HOM	−5.28	0.60			
			**APE**	**AUS**	**HOM**
**angle from the origin**			26.5	68.24	73.53

The row names correspond to individual comparisons of one group to another. APE = great apes exclusive of hominins, AUS = australopiths, HOM = *Homo* species. Calculations were performed on the whole numbers, while the table only reports approximated round-down values to 2dp.

### Mandibular shape evolution, dental occlusion, and canine size

Our results show that mandibular shape in hominins evolved faster than in any other primate clade. Contrary to our expectations, the rate of evolution in *Homo* is not smaller than in the australopiths, and the direction of the shape change velocity is one and the same for the two hominin clades. This means that the reason for the unexpected pattern of rapid mandible shape evolution observed across hominins has to be found among the characteristics shared by the australopiths and *Homo*. According to a large corpus of available data, the australopiths and *Homo* differ from each other in terms of habitat preferences, body size, patterns of sexual dimorphism, diet and food processing behaviour^16,17^. However, tool use has been hypothesized to occur in all early hominids, including australopiths^18–20^. Such emphasis on mechanical food processing might have caused parallel evolutionary changes in the mandible of hominins. Relevant dental features shared by all hominins are the reduction of maxillary canines crown height, reduced sexual dimorphism^21^, and loss of the honing capacity of the C/P_3_ complex^22^, which by contrast represents a nearly ubiquitous and stable adaptation in nonhuman anthropoids. As compared to the greater apes, all hominins evolved after *A*. *anamensis* also share a derived temporomandibular joint^23^, that allows for a peculiar forward translation and rotation of the mandible during mouth opening in increase gape^24,25^, and show strongly reduced anterior dentition (incisors and canines), shorter mandibular corpus with more divergent rami and an increase in the absolute and relative size and complexity of the post-canine dentition. The evolutionary emergence of these features has been related to dietary shifts, sexual selection, or a combination of both^26,27^. Stelzer *et al*.^28^ suggest that the reduction in incisors size, and the assumption of the parabolic dental arcade in *Homo* was due to canine and diastema reduction, rather than being selected per se. In turn, whereas usually interpreted as evolving under sexual selection, canine size in male hominins is functionally linked to an increase in mechanical efficiency of the jaws, in order to preserve gape and bite force^21,29–31^. Hylander^21,30^ argued that in hominins feeding on tough foods items bite force is increased by a forward shift in the position of the jaw muscles. Yet, this comes at the cost of decreasing gape. The reduced gape thus becomes incompatible with vertically elongated canines, hence with a working C/P_3_ honing complex^21,30^, because the P_3_ has to slide forward towards the canine tip, rather than producing sliding friction against the upper canine rear margin. However, there is no evidence that the earliest hominins such as *Sahelanthropus*, *Ardipithecus* and *A*. *anamensis*, which all show a non-honing C/ P_3_ complex, were tough food consumers^4,26,32,33^. Hylander^30^ found that among the living catarrhines intersexual differences in the degree of canine overlap and gape are not significant only in *Homo sapiens* and the hylobatids. Inspired by these reports, we repeated the multivariate angle calculation taking lesser apes in consideration. Intriguingly, whereas the trajectories of the two hominin groups remain parallel, and both are significantly or marginally different from the trajectory of the other great apes, hylobatids are not smaller (in multivariate angle) than either hominins or great apes (Tables 2, S3). Delezene^31^ showed that since the inception of our own clade (i.e. with the appearance of *Sahelanthropus*, *Orrorin*, and *Ardipithecus*) there was no longer any integration or covariation either between the canines and third lower premolars, which is necessary for efficient honing. While this might have served to increase bite force in early hominins^34,35^, its most important evolutionary consequence could have been the increased evolvability of premolars and increased pattern of reduction of the anterior dentition, including incisors. Such rapid evolution in the dentition (hence in mandible shape) has profound adaptive significance^36^. It might have permitted the acquisition, in the later species, of deep mandibular corpus and strong ramus^25,37^ in relationship to though food consumption^7,38^. Differences in absolute size and relative position of the cheek teeth link to major changes in the trophic niches of our ancestors during the Plio-Pleistocene^4,9^, and to the ever more extensive use of stone tools.

**Table 2:** Multivariate angle of evolutionary rates.

comparisons	Difference in angle	p.value			
APE_AUS	−29.05	0.07			
APE_HOM	−45.17	0.01			
APE_HYLO	−26.33	0.06			
AUS_HOM	−16.11	0.24			
AUS_HYLO	2.72	0.54			
HOM_HYLO	18.84	0.85			
		**APE**	**AUS**	**HOM**	**HYLO**
**angle from the origin**		69.26	98.31	114.43	95.59

The row names correspond to individual comparisons of one group to another. APE = great apes exclusive of hominins, AUS = australopiths, HOM = *Homo* species, HYLO = lesser apes. Calculations were performed on the whole numbers, while the table only reports approximated round-down values to 2dp.

Even if many aspects of mandibular and dental morphology, as for example the high rami in the mandible of the lineage *A*. *afarensis – P*. *boisei* and the development of megadontia in the *Paranthropus* are functionally related with some major shift in diet, it is unlikely that food adaptations per se may account for the high rates of mandible shape evolution along the entire hominin lineage. Taking in consideration the differences in both dietary and food processing habits between the australopiths and *Homo*, the vectors of the rates should be divergent, which we found was not the case. Intriguingly, sexual selection cannot explain the very high rates we observed in *Homo sapiens* and *Homo neanderthalensis* that are the species showing the lowest level of sexual dimorphism among primates, and the ostensibly divergent shape in *Homo sapiens* mandible is not shared by the Neanderthals^36,39^.

We propose the reshaping of the mandible, shared by the australopiths and *Homo*, was startled by both biomechanical and “structural” events such as the loss of a functioning of the C/P_3_ honing complex^22^. This exaptive condition occurred early in hominin evolution and generated “cascading effects” that were recruited for a number of different adaptations along and across the history of the human clade, in response to the rapid environmental changes recorded in Africa from the Upper Miocene through the Plio-Pleistocene.

## Methods

### Geometric Morphometrics of Primate mandibles

We used Geometric Mophometrics (Gmm^40,41^) to extract morphological data. This method permits to retrieve shape information of anatomical objects after removing non-shape variation (i.e. as related to size, position and orientation of the objects) by applying Generalized Procrustes Superimposition (GPA^42^). By using the TpsRelw software ver. 1.53 we performed Relative Warps Analysis on aligned coordinates (RWA^43^) to decompose shape variation into orthogonal axes of maximum variance.

For this study we collected (either by taking pictures directly, from digital sources, or from published pictures) 731 digital images of primate hemimandibles, belonging to 211 species (148 extant, 63 extinct). The number of mandibles per species ranges from 1 to 13 (median = 3, mean = 3.48). The requirements for picture inclusion in the dataset were the presence of anatomical regions where landmarks had to be placed, absence of distortions and breakages on the bone, and orientation perpendicular to the picture plane. Fortunately, being the hemimandible a flat bone, these features were easily recognizable, even on samples taken from published resources. The pictures we took directly derive from ref.^2^. We used tpsDig2 software to digitize 9 landmarks as to adequately describe the lower jaw profile (fig. S4). Gmm also returns the Centroid Size (the square root of the sum of squared distances between each landmark and the centroid of each configuration), a metric that permits to get back the information related to size that are removed by GPA. We regressed the natural logarithm of centroid size (lncs) and ln body mass estimates taken from the literature, to assess whether lncs works good as a proxy for body size. The regression is highly significant and positive (slope = 0.300, R^2^ = 0.844, p < 0.001, fig. S5). Shape variance was decomposed into 14 axes (Relative Warps). We performed the Gmm analyses twice: on the full dataset, and on a dataset deprived from pictures we obtained from literature. The former dataset (FULL) consists of 211 species, the reduced dataset (SMALL) includes pictures for 158 species (145 extant, 13 extinct). For both dataset, we used for the rate analyses only the four first largest RW axes, as they capture some 90% of the shape variance.

### RRphylo

The Phylogenetic Ridge Race Regression version we present here (‘RRphylo’) develops on phylogenetic ridge regression as described in^15^. It applies penalized ridge regression to the tree and species data. The difference between the phenotype at each tip and the phenotype at the tree root is the sum of a vector of phenotypic transformations along the root to tip path, given by equation (1)

1 $${\Delta }P={\beta }_{{1}}{{1}}_{{1}+}{\beta }_{{2}}{{1}}_{{2}+}{\mathrm{...}}_{+}{\beta }_{n}{{1}}_{n}$$

where the *β*_*ith*_ and *l*_*ith*_ elements represent the regression coefficient and branch length, respectively, for each *i*_*th*_ branch along the path. As regression slopes, the *β* coefficients represent the actual rate of phenotypic transformation along each branch. The matrix solution to find the vector of *β* coefficients for all the branches is given by equation (2) ref.^44^;

2 $$\hat{\beta }={({{\bf{L}}}^{T}{\bf{L}}+\lambda {\bf{I}})}^{-1}{{\bf{L}}}^{T}{\rm{y}}$$

where **L** is the matrix of tip to root distances of the tree (the branch lengths), having tips as rows. For each row of **L**, entries are zeroes for branches outside the tip to root path, and actual branch lengths for those branches along the path. The vector $\hat{y}$ is the vector of phenotypes (tip values), $\widehat{\beta \,}$ is the vector of regression coefficients, and *λ* is a penalization factor that avoids perfect predictions of $\hat{y}$, therefore allowing for the estimation of the vector of ancestral states, computed as in equation (3):

3 $$\hat{a}={\bf{L}}{\boldsymbol{^{\prime} }}\hat{\beta }$$

where ${{\bf{L}}}^{\text{'}}$ is the node to root path matrix, calculated in analogy to **L**, but with nodes as rows.

After computing the rates for the tree branches, we searched for shifts in the rates across the tree. This rate by clade (RBC) analysis within RRphylo scans the tree to find shifts in the rate of phenotypic evolution. There are a number of methods available in literature to apply model-free computations of the evolutionary rates^45^, yet some of them do not work with fossil phylogenies (e.g. ref.^46^) or are computationally very intensive. With RRphylo, the Brownian rate (σ^2^) is calculated for all clades as large as the user specifies (in terms of number of tips). Individual nodes (i.e. the clade they subtend to) are arranged according to their rates (i.e. in descending σ^2^ value). Then, the user is left with two different options to locate a number of potential shifts. First, it is possible to specify the number *n* of shifts to be searched for all combinations of the *n* clades with the *n* largest σ^2^ value, with size 1 to *n*. For instance, with *n* = 3 RRphylo will search through all the eight possible combinations of the 3 nodes with the largest σ^2^ values (three combinations with one shift only, one for each node; three combinations of two shifts at two different nodes; and a single combination including all the three shifts for all *n* = 3 nodes, plus Brownian motion, which means no shift applied). Alternatively, all selected nodes are partitioned in groups according to their patristic distance, and the number of distinct groups with potential shifts is established via bootstrapped cluster analysis of the internodes distances. This way the number of potential shifts are located in topologically distinct parts of the tree. The resulting number of groups *k* is thus taken to be equivalent to the number of shift to be searched, by examining all possible combinations of the *k* nodes with the largest σ^2^ values. Of course, it is still possible (and in fact tested) that more than one shift fall in the same region of the tree.

Once potential shifts are located, their combinations represent different rate variation models, which are compared to each other (and to a single rate, pure Brownian motion model) by means of restricted maximum likelihood fitted with the function brownieREML in phytools^47^, in the case of a single variable, or mvBM in mvMORPH^48^ in the multivariate case. The likelihoods of individual models are contrasted to each other to find the best model by means of likelihood ratio test. It is important to note that whereas RRphylo assigns each branch its own rate of evolution, shifts are located by assessing the likelihood of multi-rate Brownian motion models.

### Accounting for phylogenetic uncertainty in node age and topology

The distribution of evolutionary rates depends on the distribution of branch lengths and on the tree topology^49^. Every phylogenetic tree represents at best a phylogenetic hypothesis, which should be evaluated against alternative topologies, and branch lengths. To account for phylogenetic uncertainty, we wrote an Rcode that changes the tree topology and branch lengths. For every given species, the function swaps the phylogenetic position up to two nodes distance. For instance, the topology ((A,(B,C)),D) could be swapped to the forms ((C,D),(A,B)); (((B,D),A),C) and so on. In addition, each node age is randomly set at any age between the age of its parental node, and the age of its oldest daughter node. We applied the tree swapping function 100 times, computed RRphylo rates at each time, and draw the difference in mean absolute rates between the human clade and the rest of the tree each time.

### Multivariate angle computation of evolutionary rates

Our goal was to verify whether the shape trajectory in *Homo* and australopiths were parallel, and whether they differed from that of non-hominin apes. One limitation with traditional trajectory analysis (e.g. ref.^50^) is that it ignores phylogenetic relationships. To overcome this problem, we analysed shape trajectories by using phylogenetic ridge regression results.

In the context of RRphylo, each branch of the tree has its own rate vector computed. With our data, such rate is composed by the *β* coefficients of individual RW scores. The magnitude of the rate vector (i.e. the evolutionary rate) is equivalent to the square root of the sum of squared *β* coefficients. Direction is defined in reference to another vector, computing the angle between the two. Assuming **A** and **B** are two rate vectors the angle between them $\,\theta$ is defined by equation (4):

4 $$\theta =arc\,\cos \,\frac{A\cdot B}{|A||B|}$$

Thus, the path between any node in the tree and a given tip is given by the trigonometric addition of successive vectors, aligned along the node to tip path, which could be summarized as a resultant vector having its own magnitude and angle to the node. For instance, given a species and two successive parental nodes above it, so that the node-to-species path sequence is Node1/Node2/species, the resultant vector $\,\overrightarrow{R}$ is given by equation (5):

5 $$\overrightarrow{R}={\overrightarrow{A}}_{Node1}+{\overrightarrow{B}}_{Node2}+\,{\overrightarrow{C}}_{species}$$

$\overrightarrow{R}$ is centered on Node1, so that $\overrightarrow{R}\,$ will be at a certain angle to it. Here, we computed the angle between each ape species and the most recent common ancestor common to all of them (the species to apes most recent common ancestor angles) and contrasted the angles between species partitioned into non-hominin great apes (just ‘apes’ for simplicity), species belonging to *Homo*, and the australopiths. We measured the difference in mean angles between groups and generated a family of 10,000 random differences by shuffling angles between individual species. If the actual mean angle difference between two groups is larger than expected by chance, it means that the between groups trajectories are divergent, otherwise they are parallel.

## Electronic supplementary material

Supplementary Material
