# Supplementary material for: Unexpectedly rapid evolution of mandibular shape in hominins
Source: Sci Rep. 2018 May 9;8:7340. doi: 10.1038/s41598-018-25309-8 (PMC5943523; doi:10.1038/s41598-018-25309-8)
Supplement: Supplementary file 1 — Supplementary Material [file 41598_2018_25309_MOESM1_ESM.pdf]

Supplementary Material to:

Unexpectedly rapid evolution of mandibular shape in hominins

Raia P., Boggioni M., Carotenuto F., Castiglione S., Di Febbraro M., Di Vincenzo F., Melchionna M., Mondanaro A., Papini A., Profico A., Serio C., Veneziano A., Vero V.A., Rook L., Meloro C., Manzi G.

The Primate Tree

To build the Primate tree we started by downloading the consensus Bayesian inference phylogeny of extant primates from the 10KTrees website. We removed extant species we had no mandible shape data for from this tree, and then added fossil taxa we had mandible shape data, by relying on published accounts of their phylogenetic position and age. Our main reference for high-level relationships of extinct primates was the phylogenetic analysis of Pattison et al. (2014). Further topological and chronological inference about early "plesiadapiforms" and early strepsirrhines were taken from Chester and Bloch (2013), Silcox et al. (2009), and Franzen et al. (2009). Similarly, we supplemented the phylogenetic and age information on anthropoids following Ducrocq (2001), Ross et al. (1998), Gunnell and Miller (2001), Bajpai et al. (2008), and Jaeger (1998); on apes following Harrison (1986), Begun (1994), Begun and Gulec (1998), Moyà-Solà et al. (2009), Alba et al. (2010); on hominins following Dembo et al. (2015); on New World monkeys following Guedes and Salles (2005), and Horovitz and MacPhee (1999); on early catarrhines following Thomas (1991), Benefit (1999), and Williams et al. (2007); and on lemurs after Herrera and Davalos (2016), and Godfray and Jungers (2010). Additional, fossil calibration dates were taken from Pozzi et al. (2014). Each tip (species) age was set at the species last occurrence in the fossil record, as detailed in the supplementary material. By using tip ages and calibrated nodes, we assigned unknown node ages

applying the calibration method in Brusatte et al. (2008). The resulting tree includes 153 extant and 58 extinct species. The number of fossil species is limited by the availability of realistically complete fossil primate mandibles. Nonetheless, we strove to maintain temporal and phylogenetic homogeneity in sampling. Therefore, the tree includes 4 Paleocene, 16 Eocene, 5 Oligocene, and 7 Miocene species, which is reasonable given the great diversity of Eocene primates (Gingerich 1977, Jaeger et al., 2010), and the well-known paucity of the Oligocene primate record (Tavaré et al.

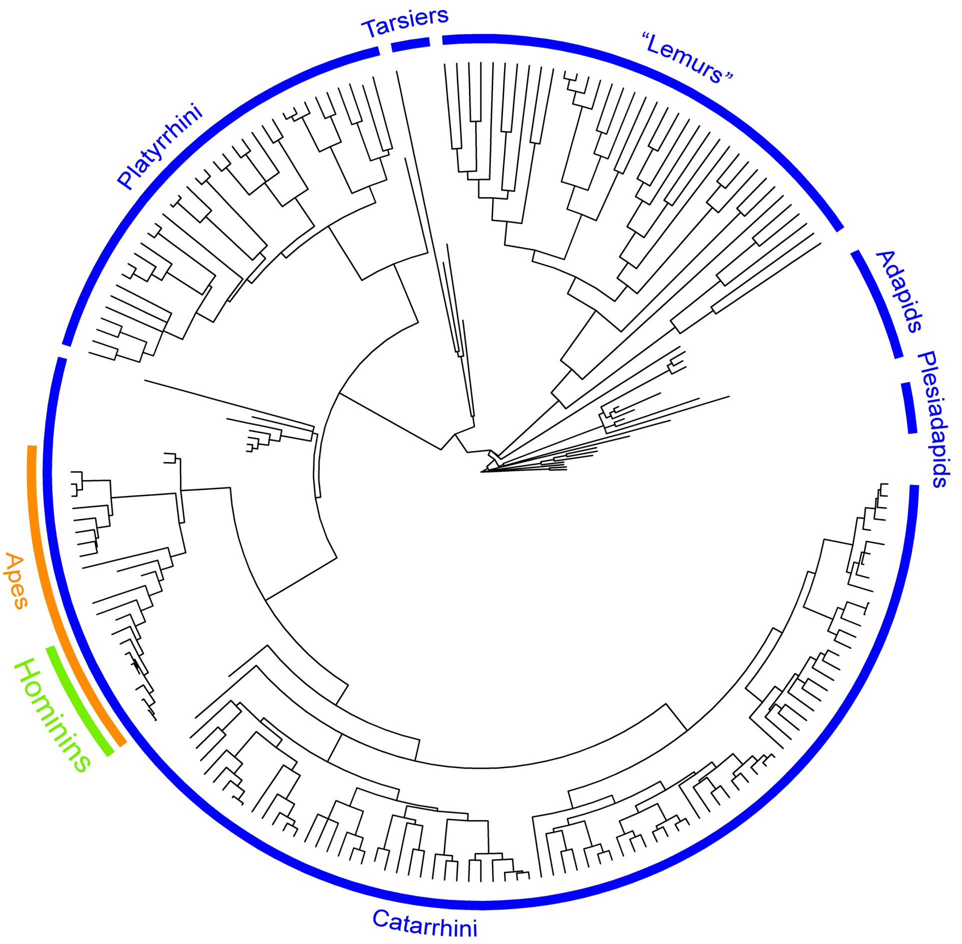

2002). One hundred fifty-five haplorrhines, and 32 lemuriformes are present in the tree, the remaining taxa belong to either adapiformes or "plesiadapiformes".

fig S1 – The composite Primate phylogenetic tree used in this study

Phylogenetic tree for the FULL dataset (211 species)
((Dryomomys_szalayi:3.20408101,Tinimomys_tribos:3.20408101):13,((Ignacius_frugivorus:13.20
408,(Picrodus_lepidus:4.30408,((Plesiadapis_cookei:5.86047356,Plesiadapis_tricuspidens:5.86047
356):2.5,Plesiadapis_rex:7.36047356):2.5):9):2,(((Darwinius_masillae:32.60408101,(((Smilodectes
_gracilis:4.48199628,(Cantius_torresi:9.28199628,Cantius_abditus:1):1):1,((Magnadapis_intermedi
us:2,Adapis_parisiensis:3.23934609):1,Protoadapis_weigelti:3):14.78199628):1,(Wailekia_oriental
e:16.7,Marcgodinotius_indicus:2):2.08199628,Aframonius_dieides:24.28199628):20.32208473)Ad
apidae:1,(Plesiopithecus_teras:40.00408101,((((((((((((Cercopithecus_albogularis:2.491503,Cercop
ithecus_mitis:2.491503):1.271137,Cercopithecus_nictitans:3.76264):2.666569,((Cercopithecus_asc
anius:2.018943,Cercopithecus_cephus:2.018944):0.712693,(Cercopithecus_erythrogaster:1.347404,
Cercopithecus_petaurista:1.347404):1.384232):3.697573):1.461009,((((Cercopithecus_campbelli:2.
247911,Cercopithecus_mona:2.247911):0.919265,((Cercopithecus_pogonias:0.885679,Cercopithec
us_denti:0.885679):0.885679,Cercopithecus_wolfi:1.771358):1.395818):2.820105,((Cercopithecus
_diana:2.794255,Cercopithecus_roloway:2.794255):2.794255,Cercopithecus_neglectus:5.588511):
0.39877):1.355876,Cercopithecus_hamlyni:7.343158):0.54706):1.957908,(((Cercopithecus_lhoesti:
4.08365,Cercopithecus_solatus:4.08365):3.85615,Erythrocebus_patas:7.9398):0.687445,((Chloroce
bus_aethiops:1.880772,Chlorocebus_pygerythrus:1.880772):1.687586,Chlorocebus_sabaeus:3.568
358):5.058886):1.220881):1.644988,Miopithecus_ogouensis:11.49417601):3.382065,((((Cercocebu
s_galeritus:3.998505,(Cercocebus_torquatus:0.172462,Cercocebus_atys:0.172462):3.826043):1.305
793,(Mandrillus_leucophaeus:3.415204,Mandrillus_sphinx:3.415204):1.889093):6.046166,((Lopho
cebus_albigena:4.896861,((Theropithecus_baringensis:0.402075992,Theropithecus_brumpti:1.1279
24008,Theropithecus_gelada:3.396862):0.5,Theropithecus_oswaldi:2.497924008):1):0.872302,((((
Papio_anubis:1.277228,Papio_hamadryas:1.277228):0.432578,Papio_papio:1.709806):0.34864,Pap
io_cynocephalus:2.058446):1.858106,Parapapio_whitei:1.422384992):1.352611):5.5813):1.502062
,(((((Macaca_arctoides:3.705214,Macaca_anderssoni:2.306276008):1,Macaca_sinica:4.705214):1.3
31851,(Macaca_nigra:4.267136,Macaca_silenus:4.267135):1.76993,Macaca_fuscata:6.037065):0.8
40931,((Macaca_fascicularis:5.045978,Macaca_mulatta:5.045978):1.475126,Macaca_thibetana:6.5
21104):0.356891):1.782804,Macaca_sylvanus:8.6608):4.191726):2.022653):6.535196,(((((Colobus
_angolensis:3.71468,(Colobus_guereza:2.306768,(Colobus_polykomos:0.339509,Colobus_velleros
us:0.339509):1.967259):1.407911):2.508901,Colobus_satanas:6.22358):6.313872,((Piliocolobus_b
adius:5.435125,(Piliocolobus_kirkii:2.646772,(Piliocolobus_rufomitratus:1.599744,Piliocolobus_th
olloni:1.599744):1.047028):2.788352):5.212325,Procolobus_verus:10.64745):1.890002):1.897705,
((Paracolobus_chemeroni:5.75,Rhinocolobus_turkanaensis:6.810000002):5.236220008,Cercopithec
oides_meaveae:10.43622001):1):1,((((Nasalis_larvatus:9.634813,(Pygathrix_nemaeus:5.001557,Py
gathrix_nigripes:5.001557):4.633255):0.839771,(Rhinopithecus_lantianensis:2.637714008,Rhinopi
thecus_avunculus:3.786652):6.687932):2.310063,(((Semnopithecus_entellus:3.654042,Trachypithe
cus_johnii:3.654042):1.01944,Trachypithecus_vetulus:4.673482):6.787895,((((Trachypithecus_aur
atus:1.269645,Trachypithecus_germaini:1.269645):0.309244,Trachypithecus_cristatus:1.57889):2.4
36117,Trachypithecus_barbei:4.015007):1.23798,(Trachypithecus_francoisi:4.023264,Trachypithec
us_phayrei:4.023264):1.229723):6.208391):1.32327):0.523455,((Presbytis_melalophos:5.778211,P
resbytis_rubiconda:5.778211,Presbytis_siamensis:5.778211):3.764946,((Presbytis_frontata:2.38579
,Presbytis_chrysomelas:2.38579):2.38579,Presbytis_hosei:4.771579):4.771579):3.764946):2.12705
6):5.975217,Mesopithecus_pentelicus:15.21143701)Cercopithecoidea:4.089626,Victoriapithecus_
macinnesi:9.401063008):4.5,(((((((Hylobates_moloch:3.309516,Hylobates_muelleri:3.309516):0.21
945,Hylobates_lar:3.528966):0.547859,Hylobates_pileatus:4.076825):2.521536,Symphalangus_syn
dactylus:6.598362):0.749537,(Nomascus_concolor:2.012645,(Nomascus_gabriellae:0.944453,Nom
ascus_leucogenys:0.944454):1.068192):5.335253):12.258047,((((Gorilla_gorilla:10.476233,((((((((
Homo_neanderthalensis:0.16,Homo_sapiens:0.2):0.4,Homo_heidelbergensis:0.4):1.4,Homo_erectu

s:1.7):1,((Homo_habilis:0.5,Homo_naledi:0.1):0.25,Australopithecus_sediba:0.85):0.25):1,Australo
pithecus_africanus:2.1):1,Paranthropus_boisei:3.8):0.9,Australopithecus_afarensis:2.9):2.1,Pan_tro
glodytes:7.999999):2.476353):2.656102,Dryopithecus_brancoi:2.783517):1,Oreopithecus_bambolii
:7.933517008):2.473491,Pongo_pygmaeus:16.605946):3):1,(Proconsul_heseloni:2.1,Proconsul_nya
nzae:2.1):1)Hominoidea:9.394055):15.811821,(Pliopithecus_conmatensis:33.21288401,((((Apidiu
m_phiomense:1,Parapithecus_grangeri:1):1,Abuqatrania_basiodontos:2):2,Arsinoea_kallimos:4):2,
Catopithecus_browni:10.6):5.812884008,Aegyptopithecus_zeuxis:11.81288401)'pithecoids':1)Catar
rhini:1,(Homunculus_patagonicus:18.41288401,((((Alouatta_belzebul:3.888217,Alouatta_seniculus
:3.888216):10.872024,(Caipora_bambuiorum:9.138454008,(Ateles_belzebuth:2.680476,Ateles_pan
iscus:2.680475):6.466916,(Brachyteles_arachnoides:2.35377,Lagothrix_lagotricha:2.35377):6.7936
21):5.612849):6.56106,((Aotus_azarae:19.487522,(((((Callithrix_argentata:1.73192,Callithrix_hum
eralifera:1.731919):3.057892,Callithrix_pygmaea:4.789812):2.239318,Callithrix_jacchus:7.02913):
8.000934,(Leontopithecus_chrysomelas:1.45399,Leontopithecus_chrysopygus:1.45399):13.576075
):0.682187,(Saguinus_inustus:11.72614,Saguinus_labiatus:11.72614,((Saguinus_bicolor:4.242081,
Saguinus_midas:4.242081):5.42139,(Saguinus_imperator:5.012547,Saguinus_mystax:5.012547):4.
650925):2.06267,Saguinus_fuscicollis:11.726141):3.986111):3.775269):0.605006,(((Cebus_albifro
ns:0.64113,Cebus_olivaceus:0.64113):5.808254,Cebus_apella:6.449384):12.120521,(Saimiri_sciur
eus:1.656503,Saimiri_ustus:1.656503):16.913402):1.522623):1.228773):1.411478,(((Cacajao_calv
us:2.274028,(Chiropotes_albinasus:1.274028,Chiropotes_satanas:1.274028):1):7.368851,(Pithecia_
monachus:4.404566,Pithecia_irrorata:4.404566):5.238313):9.753209,((Antillothrix_bernensis:13.9
9715001,Paralouatta_varonai:12.99715001):1,(Callicebus_moloch:7.360073,Callicebus_personatus
:7.360074):7.636014):4.4):3.336691)Platyrrhini:12.079043):12)Anthropoidea:22.028211,(Archiceb
us_achilles:18.2411,((Omomys_carteri:17.1,Tarsius_tarsier:54.29893599):1,Eosimias_centennicus:
21.4):11.54003):2)Haplorrhini:4.162986,((((Nycticebus_coucang:9.665419738,Nycticebus_pygmae
us:9.665419361):9.790378029,Loris_tardigradus:19.45579739):12.15699407,(Arctocebus_calabare
nsis:23.9235999,Perodicticus_potto:23.9235999):7.689192815):25.86193922,((((((Lepilemur_edwa
rdsi:6.637754958,Lepilemur_ruficaudatus:6.637754519):4.336452246,Lepilemur_dorsalis:10.9742
0739):21.67410611,(Phaner_furcifer:29.04135939,((Cheirogaleus_medius:15.46912778,Cheirogale
us_major:15.4691259):7.985212736,(((Microcebus_murinus:8.143206591,Microcebus_rufus:8.143
206115):6.475598094,Mirza_coquereli:14.61880155):3.085775202,Allocebus_trichotis:17.704575
5):5.749761886):5.587020128):3.606952223):3.812019197,((((Lemur_catta:13.5357503,(Hapalem
ur_griseus:9.257548571,Hapalemur_simus:9.257550202):4.278199473):6.95432819,(((Eulemur_al
bifrons:1.318481143,Eulemur_fulvus:1.318480829):1.91382626,Eulemur_cinereiceps:3.23230708
9):2.459661883,Eulemur_mongoz:5.6919696):14.79811203):4.649877796,(Varecia_variegata:15.0
0230446,Pachylemur_insignis:15):10.13764995):9.150383621,(((Avahi_laniger:23.98515889,(Prop
ithecus_diadema:12.92449488,Propithecus_verreauxi:12.92449614):11.0606615):1.611236567,(In
dri_indri:22.13337469,((Palaeopropithecus_ingens:11.13953703,Archaeoindris_fontoynontii:11.13
827289):9.408494351,(Babakotia_radofilai:16.1240284,Mesopropithecus_globiceps:16.12783023):
4.419763253):1.584238518):3.463019512):4.220787998,(Archaeolemur_majori:14.40441819,Hadr
opithecus_stenognathus:14.4041936):15.41122194):4.473157706):2.169989647):3.516325507,(Me
galadapis_edwardsi:7.975528722,Megaladapis_grandidieri:7.975528721):32.00002155):11.565540
19,Daubentonia_madagascariensis:51.54219463)Lemuridae:5.932532289):15.52934531):1):2,Teilh
ardina_asiatica:27.40408101):1):0.5187777887);

Phylogenetic tree for the SMALL dataset (158 species)

(Plesiadapis_rex:20.86047356,((Smilodectes_gracilis:6.48199628,Aframonius_dieides:24.2819962
8):21.32208473,(((((((((((((Cercopithecus_albogularis:2.491503,Cercopithecus_mitis:2.491503):1.2
71137,Cercopithecus_nictitans:3.76264):2.666569,((Cercopithecus_ascanius:2.018943,Cercopithec
us_cephus:2.018944):0.712693,(Cercopithecus_erythrogaster:1.347404,Cercopithecus_petaurista:1.
347404):1.384232):3.697573):1.461009,((((Cercopithecus_campbelli:2.247911,Cercopithecus_mon
a:2.247911):0.919265,((Cercopithecus_pogonias:0.885679,Cercopithecus_denti:0.885679):0.88567
9,Cercopithecus_wolfi:1.771358):1.395818):2.820105,((Cercopithecus_diana:2.794255,Cercopithe
cus_roloway:2.794255):2.794255,Cercopithecus_neglectus:5.588511):0.39877):1.355876,Cercopit
hecus_hamlyni:7.343158):0.54706):1.957908,(((Cercopithecus_lhoesti:4.08365,Cercopithecus_sola
tus:4.08365):3.85615,Erythrocebus_patas:7.9398):0.687445,((Chlorocebus_aethiops:1.880772,Chlo
rocebus_pygerythrus:1.880772):1.687586,Chlorocebus_sabaeus:3.568358):5.058886):1.220881):1.
644988,Miopithecus_ogouensis:11.49417601):3.382065,((((Cercocebus_galeritus:3.998505,(Cerco
cebus_torquatus:0.172462,Cercocebus_atys:0.172462):3.826043):1.305793,(Mandrillus_leucophae
us:3.415204,Mandrillus_sphinx:3.415204):1.889093):6.046166,((Lophocebus_albigena:4.896861,T
heropithecus_gelada:4.896862):0.872302,(((Papio_anubis:1.277228,Papio_hamadryas:1.277228):0.
432578,Papio_papio:1.709806):0.34864,Papio_cynocephalus:2.058446):3.210717):5.5813):1.5020
62,((((Macaca_arctoides:4.705214,Macaca_sinica:4.705214):1.331851,(Macaca_nigra:4.267136,M
acaca_silenus:4.267135):1.76993,Macaca_fuscata:6.037065):0.840931,((Macaca_fascicularis:5.045
978,Macaca_mulatta:5.045978):1.475126,Macaca_thibetana:6.521104):0.356891):1.782804,Macac
a_sylvanus:8.6608):4.191726):2.022653):6.535196,((((Colobus_angolensis:3.71468,(Colobus_guer
eza:2.306768,(Colobus_polykomos:0.339509,Colobus_vellerosus:0.339509):1.967259):1.407911):
2.508901,Colobus_satanas:6.22358):6.313872,((Piliocolobus_badius:5.435125,(Piliocolobus_kirkii
:2.646772,(Piliocolobus_rufomitratus:1.599744,Piliocolobus_tholloni:1.599744):1.047028):2.7883
52):5.212325,Procolobus_verus:10.64745):1.890002):2.897705,((((Nasalis_larvatus:9.634813,(Pyg
athrix_nemaeus:5.001557,Pygathrix_nigripes:5.001557):4.633255):0.839771,Rhinopithecus_avunc
ulus:10.474584):2.310063,(((Semnopithecus_entellus:3.654042,Trachypithecus_johnii:3.654042):1
.01944,Trachypithecus_vetulus:4.673482):6.787895,((((Trachypithecus_auratus:1.269645,Trachypi
thecus_germaini:1.269645):0.309244,Trachypithecus_cristatus:1.57889):2.436117,Trachypithecus_
barbei:4.015007):1.23798,(Trachypithecus_francoisi:4.023264,Trachypithecus_phayrei:4.023264):
1.229723):6.208391):1.32327):0.523455,((Presbytis_melalophos:5.778211,Presbytis_rubiconda:5.7
78211,Presbytis_siamensis:5.778211):3.764946,((Presbytis_frontata:2.38579,Presbytis_chrysomela
s:2.38579):2.38579,Presbytis_hosei:4.771579):4.771579):3.764946):2.127056):5.975217)Cercopith
ecoidea:8.589626,(((((((Hylobates_moloch:3.309516,Hylobates_muelleri:3.309516):0.21945,Hylob
ates_lar:3.528966):0.547859,Hylobates_pileatus:4.076825):2.521536,Symphalangus_syndactylus:6
.598362):0.749537,(Nomascus_concolor:2.012645,(Nomascus_gabriellae:0.944453,Nomascus_leu
cogenys:0.944454):1.068192):5.335253):12.258047,(((Gorilla_gorilla:10.476233,(((((((Homo_nean
derthalensis:0.16,Homo_sapiens:0.2):0.4,Homo_heidelbergensis:0.4):1.4,Homo_erectus:1.7):2,Aust
ralopithecus_africanus:2.1):1,Paranthropus_boisei:3.8):0.9,Australopithecus_afarensis:2.9):2.1,Pan
_troglodytes:7.999999):2.476353):3.656102,Oreopithecus_bambolii:7.933517008):2.473491,Pongo
_pygmaeus:16.605946):3):1,Proconsul_nyanzae:3.1)Hominoidea:9.394055):15.811821,(Catopithec
us_browni:16.41288401,Aegyptopithecus_zeuxis:11.81288401)'pithecoids':1)Catarrhini:1,((((Aloua
tta_belzebul:3.888217,Alouatta_seniculus:3.888216):10.872024,((Ateles_belzebuth:2.680476,Atele
s_paniscus:2.680475):6.466916,(Brachyteles_arachnoides:2.35377,Lagothrix_lagotricha:2.35377):6
.793621):5.612849):6.56106,((Aotus_azarae:19.487522,(((((Callithrix_argentata:1.73192,Callithrix
_humeralifera:1.731919):3.057892,Callithrix_pygmaea:4.789812):2.239318,Callithrix_jacchus:7.0
2913):8.000934,(Leontopithecus_chrysomelas:1.45399,Leontopithecus_chrysopygus:1.45399):13.5
76075):0.682187,(Saguinus_inustus:11.72614,Saguinus_labiatus:11.72614,((Saguinus_bicolor:4.24
2081,Saguinus_midas:4.242081):5.42139,(Saguinus_imperator:5.012547,Saguinus_mystax:5.0125
47):4.650925):2.06267,Saguinus_fuscicollis:11.726141):3.986111):3.775269):0.605006,(((Cebus_a
lbifrons:0.64113,Cebus_olivaceus:0.64113):5.808254,Cebus_apella:6.449384):12.120521,(Saimiri_

sciureus:1.656503,Saimiri_ustus:1.656503):16.913402):1.522623):1.228773):1.411478,(((Cacajao_calvus:2.274028,(Chiropotes_albinasus:1.274028,Chiropotes_satanas:1.274028):1):7.368851,(Pithecia_monachus:4.404566,Pithecia_irrorata:4.404566):5.238313):9.753209,(Callicebus_moloch:7.360073,Callicebus_personatus:7.360074):12.036014):3.336691)Platyrrhini:24.079043)Anthropoidea:22.028211,Tarsius_tarsier:68.83896599)Haplorrhini:4.162986,((((Nycticebus_coucang:9.665419738,Nycticebus_pygmaeus:9.665419361):9.790378029,Loris_tardigradus:19.45579739):12.15699407,(Arctocebus_calabarensis:23.9235999,Perodicticus_potto:23.9235999):7.689192815):25.86193922,(((((Lepilemur_edwardsi:6.637754958,Lepilemur_ruficaudatus:6.637754519):4.336452246,Lepilemur_dorsalis:10.97420739):21.67410611,(Phaner_furcifer:29.04135939,((Cheirogaleus_medius:15.46912778,Cheirogaleus_major:15.4691259):7.985212736,(((Microcebus_murinus:8.143206591,Microcebus_rufus:8.143206115):6.475598094,Mirza_coquereli:14.61880155):3.085775202,Allocebus_trichotis:17.7045755):5.749761886):5.587020128):3.606952223):3.812019197,((((Lemur_catta:13.5357503,(Hapalemur_griseus:9.257548571,Hapalemur_simus:9.257550202):4.278199473):6.95432819,(((Eulemur_albifrons:1.318481143,Eulemur_fulvus:1.318480829):1.91382626,Eulemur_cinereiceps:3.232307089):2.459661883,Eulemur_mongoz:5.6919696):14.79811203):4.649877796,Varecia_variegata:25.13995441):9.150383621,((Avahi_laniger:23.98515889,(Propithecus_diadema:12.92449488,Propithecus_verreauxi:12.92449614):11.0606615):1.611236567,Indri_indri:25.5963942):8.693945704):2.169989647):15.0818657,Daubentonia_madagascariensis:51.54219463)Lemuridae:5.932532289):15.52934531):1):3);

Evolutionary rate analysis results

With the FULL dataset a significant rate shift applies to the clade including the genus *Homo* and the Australopiths. Potential shifts are estimated according to variation in the multivariate Brownian rate, yet the same results apply by analyzing RRphylo rates (fig. S2). We estimated whether the shift located at the clade parental to humans and the australopiths, as applied to RRphylo rates, is significant as well. To this aim, we first calculated the multivariate rate by taking the Euclidean Norm of the vector of individual variables rates (variables are the relative warp scores) per branch. Then we took the average difference between the absolute values of the multivariate RRphylo rates of the hominin clade and rates attached to the branches of the rest of the tree. Then, we assessed significance by computing 10,000 average differences obtained by randomizing rates across the tree branches. With the FULL dataset, this test indicates that the branches belonging to the hominin clade are significantly higher than background rates at $p < 0.0001$. The average multivariate rate difference is 0.065. With the SMALL dataset the average difference in the multivariate rates between the hominin clade and the rest of the tree is again significant at $p < 0.0001$, yet the computed average multivariate rate difference is slightly smaller than with the FULL dataset at 0.025.

Accounting for phylogenetic uncertainty in node age and topology

The distribution of evolutionary rates depends on the distribution of branch lengths and on the tree topology (Bapst 2014). Every phylogenetic tree represents at best a phylogenetic hypothesis, which should be evaluated against alternative topologies, and branch lengths. To account for phylogenetic uncertainty, we wrote a Rcode that changes the tree topology and branch lengths. For every given species, the function swaps the phylogenetic position up to two nodes distance. For instance, the topology ((A,(B,C)),D) could be swapped to the forms ((C,D),(A,B)); (((B,D),A),C) and so on. In addition, each node age is randomly set at any age between the age of its parental node, and the age of its oldest daughter node. We applied the tree swapping function 100 times, computed RRphylo

rates at each time, and draw the difference in mean absolute rates between the hominin clade and the rest of the tree each time. The distribution of mean absolute rate differences (fig. S3) still points to higher absolute rates in the hominin clade branches, and it is statistically significant, as assessed by means of a t-test ($t = -10.227$, $df = 11.482$, $p << 0.0001$).

	# shifted nodes	AICc	mod.likelihoods	species included	
				**node # 1**	**node # 2**
**FULL**	1	-2924.288	1486.876	*Homo neanderthalensis, Homo sapiens, Homo heidelbergensis, Homo erectus, Homo habilis, Homo naledi, Australopithecus sediba, Australopithecus africanus, Paranthropus boisei, Australopithecus afarensis*	
**FULL size free**	1	-2936.846	1493.156	*Homo neanderthalensis, Homo sapiens, Homo heidelbergensis, Homo erectus, Homo habilis, Homo naledi, Australopithecus sediba, Australopithecus africanus, Paranthropus boisei, Australopithecus afarensis*	
**FULL size and shape**	2	1375.089	-676.2225	*Cercocebus galeritus, Cercocebus torquatus, Cercocebus atys, Mandrillus leucophaeus, Mandrillus sphinx, Lophocebus albigena, Theropithecus gelada, Theropithecus baringensis, Theropithecus brumpti, Theropithecus oswaldi, Papio anubis, Papio hamadryas, Papio papio, Papio cynocephalus, Parapapio whitei*	*Cercopithecinae*
	2	1389.588	-683.4718	*Cercocebus galeritus, Cercocebus torquatus, Cercocebus atys, Mandrillus leucophaeus, Mandrillus sphinx, Lophocebus albigena, Theropithecus gelada, Theropithecus baringensis, Theropithecus brumpti, Theropithecus oswaldi, Papio anubis, Papio hamadryas, Papio papio, Papio cynocephalus, Parapapio whitei, Macaca arctoides, Macaca anderssoni, Macaca sinica, Macaca fuscata, Macaca nigra, Macaca silenus, Macaca fascicularis, Macaca mulatta, Macaca thibetana, Macaca sylvanus*	*Cercopithecinae*
**SMALL**	1	-3310.757	1618.144	*Homo neanderthalensis, Homo sapiens, Homo heidelbergensis, Homo erectus, Australopithecus africanus, Paranthropus boisei, Australopithecus afarensis*	

table S1 – Results of the multivariate rate shift test

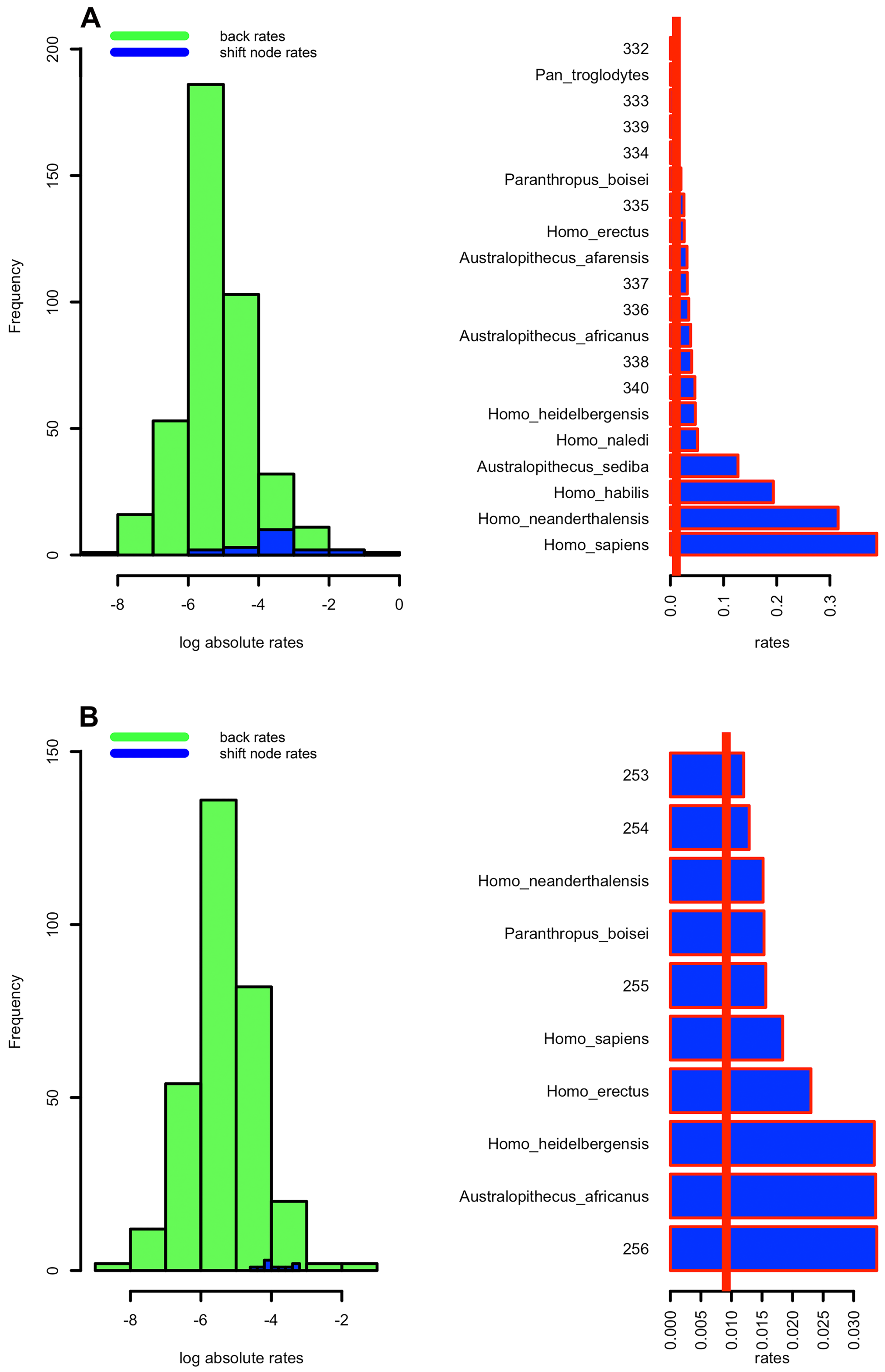

fig. S2 Left, frequency distribution of the rates calculated by RRphylo partitioned into background rates (green) and the rates of shifted nodes (blue) for the FULL dataset (A) and SMALL dataset (B). On the right, rates calculated by RRphylo for the branches belonging to the shifted node for the FULL dataset (A) and SMALL dataset (B). The red vertical line represents the average rate calculated over the entire tree.

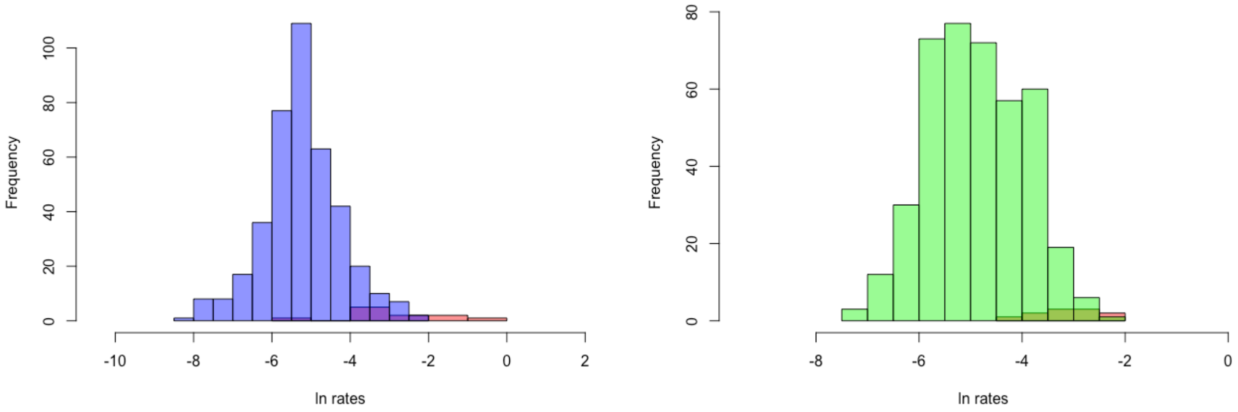

fig. S3 – Distribution of multivariate rates computed by the RRphylo method for the hominin clade as compared to the rest of the tree. The histogram to the left refers to the rates actually computed with the phylogeny of the FULL dataset (211 species). The background rates are in violet, the rates for the branches of the hominin clade are in orange. To the right, the distribution of the same rates averaged over 100 phylogenetic trees derived from the FULL dataset tree after randomly changing topology and branch lengths.

Geometric Morphometrics of Primate mandibles

We used Geometric Mophometrics (Gmm, Rohlf & Marcus, 1993; Klingenberg, 2010) to extract morphological data. This method permits to retrieve shape information of anatomical objects after removing non-shape variation (i.e. as related to size, position and orientation of the objects) by applying Generalized Procrustes Superimposition (GPA, Rohlf and Slice 1990). By using the TpsRelw software ver. 1.53 (Rohlf, 2013b) we performed Relative Warps Analysis on aligned coordinates (RWA, Bookstein 1991; Rohlf 1993; Zelditch et al. 2002) to decompose shape variation into orthogonal axes of maximum variance.

For this study we collected (either by taking pictures directly, from digital sources, or from published pictures) 731 digital images of primate hemimandibles, belonging to 211 species (148 extant, 63 extinct). The number of mandibles per species ranges from 1 to 13 (median = 3, mean = 3.48). The requirements for picture inclusion in the dataset were the presence of anatomical regions where landmarks had to be placed, absence of distortions and breakages on the bone, and orientation perpendicular to the picture plane. Fortunately, being the hemimandible a flat bone, these features were easily recognizable, even on samples taken from published resources. The pictures we took directly derive from Meloro et al. (2015).

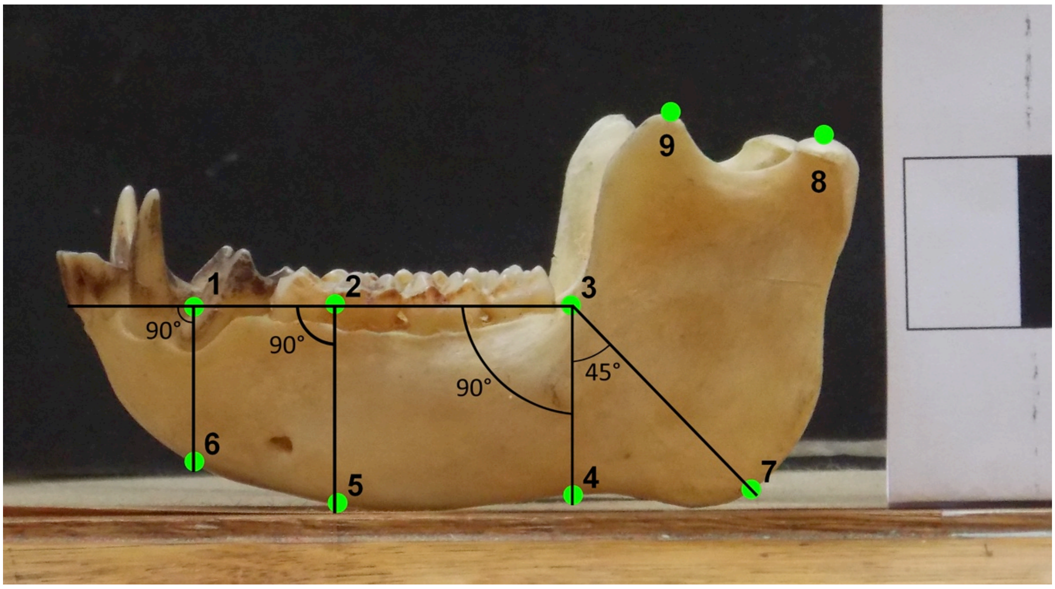

fig. S4- The Landmark configuration used in this study as applied to a Mona monkey *Cercopithecus mona* left hemimanbible. Landmarks definitions: 1) anterior tip of premolar-tooth row; 2) posterior tip of premolar-tooth row; 3) lower tip of ascending ramus; 4-5-6) intersections of the orthogonal projections of landmarks 3, 2 and 1 to the lower border of the hemimandible; 7) Intersection of the forty-five degrees projection of landmark 3 to the mandible border; 8) uppermost point of the mandibular condyle; 9) uppermost point of the mandibular coronoid.

We used tpsDig2 software (Rohlf, 2013) to digitize 9 landmarks as to adequately describe the lower jaw profile (fig. S4). Gmm also returns the Centroid Size, an index that permits to get back the information related to size that are removed by GPA. We regressed the natural logarithm of centroid size (lncs) and body mass estimates taken from the literature, to asses whether lncs works good as a proxy for body size. The regression is highly significant and positive (slope = 0.300, R^2= 0.844, $p < 0.001$, fig. S5).

Shape variance was decomposed into 14 axes (Relative Warps). We performed the Gmm analyses twice: on the full dataset, and on a dataset deprived from pictures we obtained from literature. The former dataset (FULL) consists of 211 species, the reduced dataset (SMALL) includes pictures for 158 species (145 extant, 13 extinct).

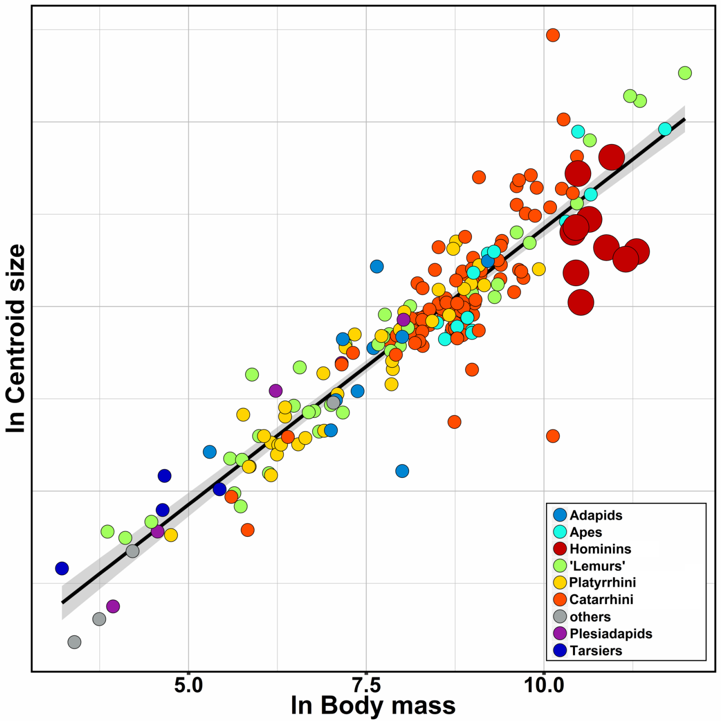

fig. S5 - The regression between the centroid size (y) and body mass (x).

With the FULL dataset, RW1 and RW2 axes describe 38.59% and 19.50% of shape variance, respectively. Positive values of RW1 are associated to jaws with slender mandibular corpus and elongated surface of attachment for the masseter. Negative values of RW2 are associated to mandibles with stronger angle and lower ramus (Fig. 1 in the main manuscript). Mandible with narrow gonial angle and high ramus occur at positive values of RW2. Specimens belonging to the same higher-level taxon show similar morphologies, except for lemurs, which spread across the morphospace. This indicates there is strong shape conservatism in primate mandibles. To confirm this hypothesis we estimated phylogenetic signal using function *physignal* (in R package Geomorph) and found it is significant ($K_{multiple}$ = 0.274, p = 0.001).

211 Full

eigenvalues %	Variance	Cumulative %
0.00743	39.44	39.44

0.00405	21.50	60.93
0.00259	13.76	74.69
0.00165	8.77	83.46
0.00097	5.15	88.61

211 Size Free

eigenvalues %	Variance	Cumulative %
0.00315	33.00	33.00
0.00237	24.88	57.88
0.00118	12.36	70.23
0.00090	9.44	79.67
0.00060	6.34	86.01

211 Size and Shape

eigenvalues %	Variance	Cumulative %
19.44259	94.34	94.34
0.39962	1.94	96.28
0.30655	1.49	97.77
0.14447	0.70	98.47
0.11897	0.58	99.05

158 Full

eigenvalues %	Variance	Cumulative %
0.00746	44.35	44.35
0.00318	18.89	63.24
0.00220	13.06	76.30
0.00149	8.87	85.16
0.00093	5.53	90.69

table S2. Results of GPA decomposition of shape for the four datasets analysed.

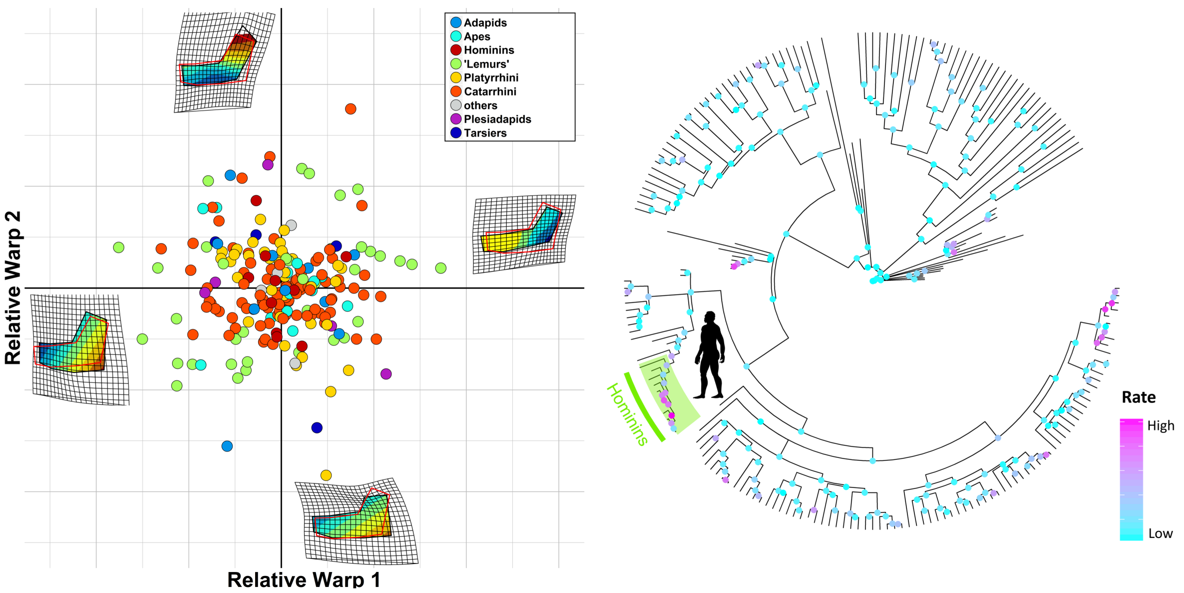

fig. S6 – GPA shape decomposition (left) and analysis of rate variation (right) in the size free shape space (without allometric effects). The clade showing a significant rate shift is highlighted with a colour box (right). Silhouettes were downloaded from Phylopic (credits as in Figure 2).

On the FULL dataset we performed a second GPA eliminating allometry, in order to account for potentially large scaling effects linked to the wide body size range in the data (Zelditch et al. 2012). To this aim, we regressed the aligned coordinates versus lncs and took the residuals to use to perform GPA. With the 'size free' dataset, the first two RWs explain 32.9% and 24.9% of the shape variance, respectively (table S2, fig. S6, S7). We additionally repeated GPA in the 'size and shape' space, and obtained 15 new axes, in particular RW1 and RW2 explain 94.35% and 2.3% of shape variance respectively (fig. S8).

Eventually, on the SMALL dataset we obtained 14 RWs. RW1 explains 44.35% and RW2 18.88% of shape variance. Along these axes the deformations are qualitatively the same that we obtained from first GPA with 211 species (fig. S9).

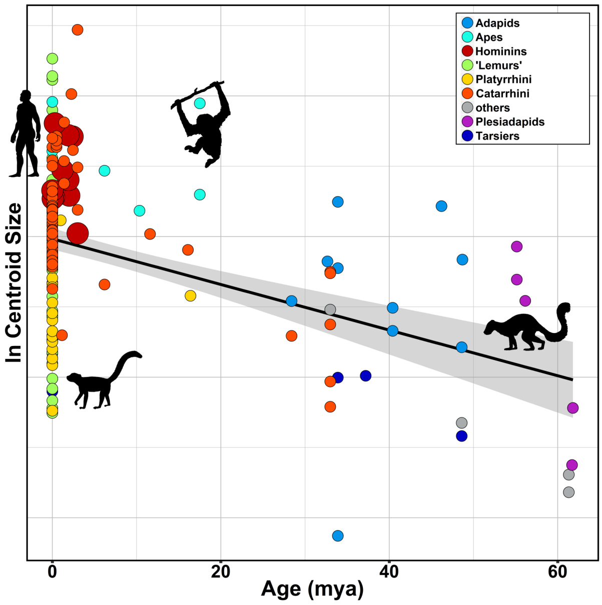

fig. S7 – Regression plot of centroid size against age. Silhouettes were downloaded from Phylopic: credits for lemuriformes ([http://phylopic.org/image/eefe8b60-9a26-46ed-a144-67f4ac885267/)](http://phylopic.org/image/eefe8b60-9a26-46ed-a144-67f4ac885267/), available for reuse under Attribution-ShareAlike 3.0 Unported ([https://creativecommons.org/licenses/by-sa/3.0/)](https://creativecommons.org/licenses/by-sa/3.0/) go to Smokeybjb; credits for *Plesiadapis* ([http://phylopic.org/image/b6ff5568-0712-4b15-a1fd-22b289af904d/)](http://phylopic.org/image/b6ff5568-0712-4b15-a1fd-22b289af904d/), available for reuse under Attribution-ShareAlike 3.0 Unported (https://creativecommons.org/licenses/by-sa/3.0/) go to Nobu Tamura (image modified by Michael Keesey).

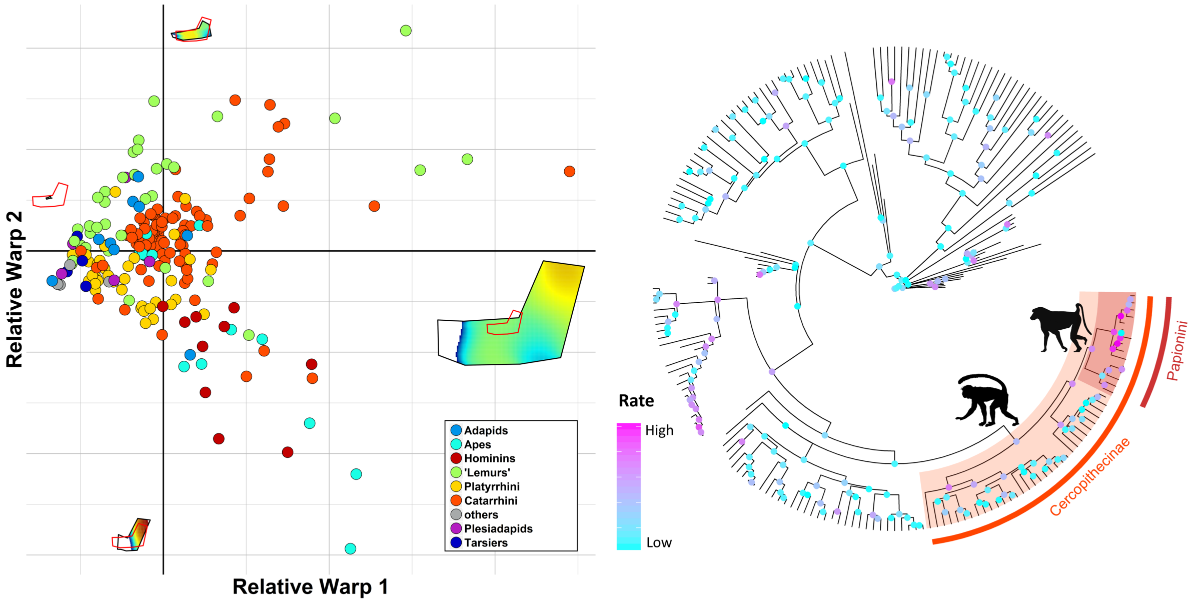

fig. S8 – GPA shape decomposition (left) and analysis of rate variation (right) in the size and shape space. Clades showing a significant rate shift are highlighted with a color box (right). Silhouettes were downloaded from Phylopic under Public Domain: *Cercopithecus* (http://phylopic.org/image/eccbb404-c99f-41f9-8785-01a7f57f1269/); *Papio* (http://phylopic.org/image/72f2f854-f3cd-4666-887c-35d5c256ab0f/).

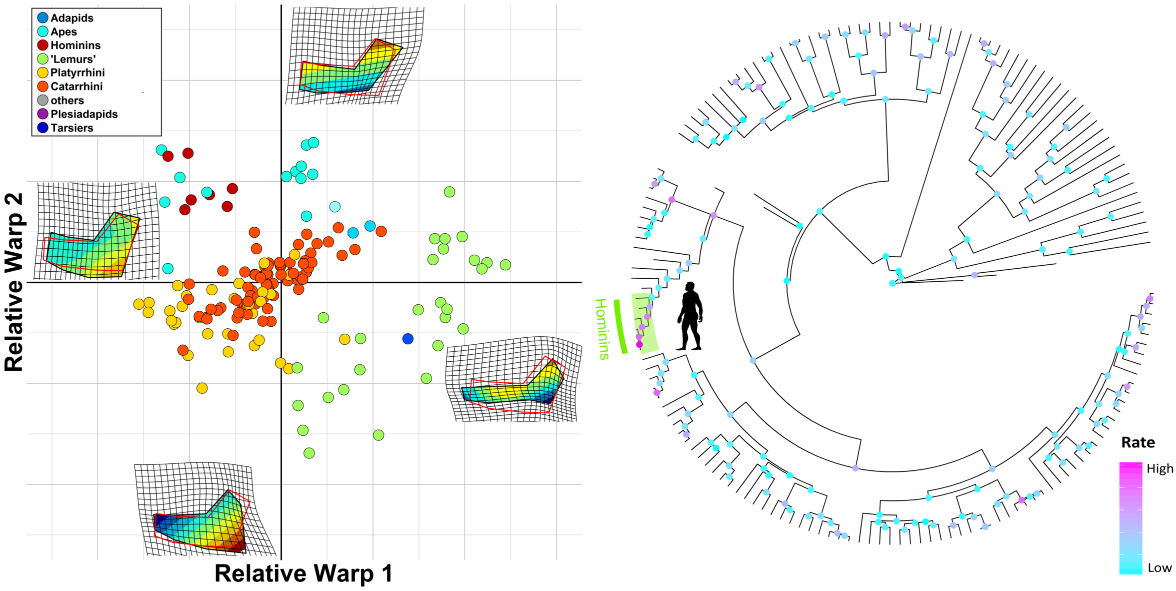

fig. S9 – GPA shape decomposition (left) and analysis of rate variation (right) in the size and shape space performed using the SMALL dataset. The clade showing a significant rate shift

are highlighted with a colour box (right). Silhouettes were downloaded from Phylopic (credits as in Figure 2).

Table S3. Multivariate Angle results, individual species

A - angles from the ancestor of all great apes

group	species	angle_from_mrca
ape	Dryopithecus_brancoi	40.332
ape	Gorilla_gorilla	34.587
ape	Pan_troglodytes	6.887
ape	Pongo_pygmaeus	24.197
aus	Australopithecus_afarensis	36.526
aus	Australopithecus_africanus	122.976
aus	Paranthropus_boisei	45.222
hom	Homo_erectus	48.625
hom	Homo_habilis	78.939
hom	Homo_heidelbergensis	66.786
hom	Homo_neanderthalensis	83.380
hom	Homo_sapiens	89.903

B - angles from the ancestor of all apes

group	species	angle_from_mrca
APE	Dryopithecus_brancoi	61.814
APE	Gorilla_gorilla	76.678
APE	Pan_troglodytes	107.036
APE	Pongo_pygmaeus	64.086
APE	Proconsul_heseloni	69.151
APE	Proconsul_nyanzae	36.779
AUS	Australopithecus_afarensis	126.771
AUS	Australopithecus_africanus	51.551
AUS	Paranthropus_boisei	116.622
HOM	Homo_erectus	130.859
HOM	Homo_habilis	149.716
HOM	Homo_heidelbergensis	114.342
HOM	Homo_neanderthalensis	124.944
HOM	Homo_sapiens	52.296
HYLO	Hylobates_lar	73.274
HYLO	Hylobates_moloch	128.053
HYLO	Hylobates_muelleri	95.934
HYLO	Hylobates_pileatus	101.629
HYLO	Nomascus_concolor	111.712
HYLO	Nomascus_gabriellae	85.880
HYLO	Nomascus_leucogenys	71.046
HYLO	Symphalangus_syndactylus	97.170

References

Alba, D. M., Fortuny, J., & Moyà-Solà, S. (2010). Enamel thickness in the Middle Miocene great apes Anoiapithecus, Pierolapithecus and Dryopithecus. *Proceedings of the Royal Society of London B: Biological Sciences*, *277*(1691), 2237-2245.

Arnold, C., L. J. Matthews and C. L. Nunn (2010). "The 10kTrees website: a new online resource for primate phylogeny." Evolutionary Anthropology: Issues, News, and Reviews 19(3): 114-118

Arnold, C., L. J. Matthews, and C. L. Nunn. 2010. The 10kTrees Website: A New Online Resource for Primate Phylogeny. *Evolutionary Anthropology*19:114-118.

Bajpai, S., Kay, R. F., Williams, B. A., Das, D. P., Kapur, V. V., & Tiwari, B. N. (2008). The oldest Asian record of Anthropoidea. *Proceedings of the National Academy of Sciences*, *105*(32), 11093-11098.

Bapst, D. W. (2014). Assessing the effect of time-scaling methods on phylogeny-based analyses in the fossil record. *Paleobiology*, 40(3), 331-351.

Begun, D. R. (1994). Relations among the great apes and humans: new interpretations based on the fossil great ape *Dryopithecus*. *American Journal of Physical Anthropology*, *37*(S19), 11-63.

Begun, D. R., & Gülec, E. (1998). Restoration of the type and palate of *Ankarapithecus meteai*: taxonomic and phylogenetic implications. *American Journal of Physical Anthropology*, *105*(3), 279-314.

Benefit, B. R. (1999). *Victoriapithecus*: the key to Old World monkey and catarrhine origins. *Evolutionary Anthropology: Issues, News, and Reviews*, *7*(5), 155-174.

Brusatte, S. L., Benton, M. J., Ruta, M. and Lloyd, G. T., 2008. Superiority, competition, and opportunism in the evolutionary radiation of dinosaurs. *Science*, 321, 1485-1488

Chester, S. G., & Bloch, J. I. (2013). Systematics of Paleogene Micromomyidae (Euarchonta, Primates) from North America. *Journal of Human Evolution*, *65*(2), 109-142.

Dembo, M., Matzke, N. J., Mooers, A. Ø., & Collard, M. (2015, August). Bayesian analysis of a morphological supermatrix sheds light on controversial fossil hominin relationships. *Proc. R. Soc. B* (Vol. 282, No. 1812, p. 20150943).

Ducrocq, S. (2001). Palaeogene anthropoid primates from Africa and Asia: new phylogenetical evidences. *Comptes Rendus de l'Académie des Sciences-Series IIA-Earth and Planetary Science*, *332*(5), 351-356.

Franzen, J. L., Gingerich, P. D., Habersetzer, J., Hurum, J. H., von Koenigswald, W., & Smith, B. H. (2009). Complete primate skeleton from the middle Eocene of Messel in Germany: morphology and paleobiology. *PLoS one*, *4*(5), e5723.

Gingerich, P. D. (1977). Radiation of Eocene adapidae in Europe. *Geobios*, *10*, 165-182.

Godfrey, L. R., Jungers, W. L., & Burney, D. A. (2010). Subfossil lemurs of Madagascar. *Cenozoic Mammals of Africa. The University of California Press, Berkeley*, 351-367.

Guedes, P. G., & Salles, L. O. (2005). New insights on the phylogenetic relationships of the two giant extinct New World monkeys (Primates, Platyrrhini). *Arquivos do Museu Nacional*, *63*(1), 147-159.

Gunnell, G. F., & Miller, E. R. (2001). Origin of Anthropoidea: dental evidence and recognition of early anthropoids in the fossil record, with comments on the Asian anthropoid radiation.

Harrison, T. (1986). A reassessment of the phylogenetic relationships of *Oreopithecus bambolii* Gervais. *Journal of Human Evolution*, *15*(7), 541-583.

Herrera, J. P., & Dávalos, L. M. (2016). Phylogeny and divergence times of lemurs inferred with recent and ancient fossils in the tree. *Systematic Biology*, *65*(5), 772-791.

Horovitz, I., & MacPhee, R. D. (1999). The quaternary Cuban platyrrhine *Paralouatta varonai* and the origin of Antillean monkeys. *Journal of Human Evolution*, *36*(1), 33-68.

Jaeger, J. J., Beard, K. C., Chaimanee, Y., Salem, M., Benammi, M., Hlal, O., ... & Valentin, X. (2010). Late middle Eocene epoch of Libya yields earliest known radiation of African anthropoids. *Nature*, *467*(7319), 1095-1098.

Jaeger, J. J., Soe, U. A. N., Aung, U. A. K., Benammi, M., Chaimanee, Y., Ducrocq, R. M., ... & Ducrocq, S. (1998). New Myanmar middle Eocene anthropoids. An Asian origin for catarrhines?. *Comptes Rendus de l'Académie des Sciences-Series III-Sciences de la Vie*, *321*(11), 953-959.

Marroig, G & Cheverud, JM (2009) Size as a line of least evolutionary resistance: diet and adaptive morphological radiation in new world monkeys. *Evolution* 59, 1128-1142.

Moyà-Solà, S., Alba, D. M., Almécija, S., Casanovas-Vilar, I., Köhler, M., De Esteban-Trivigno, S., ... & Fortuny, J. (2009). A unique Middle Miocene European hominoid and the origins of the great ape and human clade. *Proceedings of the National Academy of Sciences*, *106*(24), 9601-9606.

Pattinson, D. J., Thompson, R. S., Piotrowski, A. K., & Asher, R. J. (2014). Phylogeny, paleontology, and primates: do incomplete fossils bias the tree of life? *Systematic Biology*, 64(2), 169-186.

Pozzi, L., Hodgson, J. A., Burrell, A. S., Sterner, K. N., Raaum, R. L., & Disotell, T. R. (2014). Primate phylogenetic relationships and divergence dates inferred from complete mitochondrial genomes. *Molecular phylogenetics and evolution*, *75*, 165-183.

Ross, C., Williams, B., & Kay, R. F. (1998). Phylogenetic analysis of anthropoid relationships. *Journal of Human Evolution*, *35*(3), 221-307.

Silcox, M. T., Bloch, J. I., Boyer, D. M., Godinot, M., Ryan, T. M., Spoor, F., & Walker, A. (2009). Semicircular canal system in early primates. *Journal of Human Evolution*, *56*(3), 315-327.

Tavaré, S., Marshall, C. R., Will, O., Soligo, C., & Martin, R. D. (2002). Using the fossil record to estimate the age of the last common ancestor of extant primates. *Nature*, *416*(6882), 726-729.

Thomas, H., Roger, J., & Al-Sulaimani, Z. (1991). The discovery of *Moeripithecus markgrafi* Schlosser (Propliopithecidae, Anthropoidea, Primates), in the Ashawq Formation (Early Oligocene of Dhofar Province, Sultanate of Oman). *Journal of Human Evolution*, *20*(1), 33-49.

Williams, F. L., Ackermann, R. R., & Leigh, S. R. (2007). Inferring Plio-Pleistocene southern African biochronology from facial affinities in Parapapio and other fossil papionins. *American journal of physical anthropology*, *132*(2), 163-174.

Zelditch, M. L., Swiderski, D. L., & Sheets, H. D. (2012). Geometric morphometrics for biologists: a primer. Academic Press.
